# Use of Surgisis for Treatment of Anterior and Posterior Vaginal Prolapse

**DOI:** 10.1155/2012/376251

**Published:** 2012-01-15

**Authors:** Sara Armitage, Elvis I. Seman, Marc J. N. C. Keirse

**Affiliations:** ^1^Department of Obstetrics, Gynaecology and Reproductive Medicine, Flinders Medical Centre and Flinders University, Adelaide, SA 5042, Australia; ^2^Geralton Regional Hospital, 51-85 Shenton Street, Geralton, WA 6531, Australia

## Abstract

*Aim.* To evaluate the anatomical success and complication rate of Surgisis in the repair of anterior and posterior vaginal wall prolapse. *Methods.* A retrospective review of 65 consecutive Surgisis prolapse repairs, involving the anterior and/or posterior compartment, performed between 2003 and 2009, including their objective and subjective success rates using the pelvic organ prolapse quantification (POPQ) system. *Results.* The subjective success rate (no symptoms and no bulge beyond the hymen) was 92%, and the overall objective success rate (no subsequent prolapse in any compartment) was 66% (43 of 65). The overall reoperation rate for de novo and recurrent prolapse was 7.7% with 3 women undergoing repeat surgery at the same site (anterior compartment). No long-term complications occurred. *Conclusions.* Surgisis has a definite role in the surgical treatment of prolapse. It may decrease recurrences seen with native tissue repair and long-term complications of synthetic mesh. Its use in posterior compartment repair in particular is promising.

## 1. Introduction

The satisfactory surgical treatment of vaginal prolapse continues to elude gynaecologists, as evidenced by reports of failure rates ranging from 30% to 70% and a reoperation rate of 30% [1–3]. Permanent prostheses and mesh kits have been introduced in an attempt to improve these figures, but their use has been tempered by complications and long-term sequelae related to the techniques and materials used [4, 5].

Jia et al. [5] reviewed rates of objective failure and reoperation for failures and mesh excision of absorbable, biological, and nonabsorbable mesh in 3,000 women. For the anterior compartment, the objective failure rates for no mesh, absorbable mesh, biological grafts, and permanent mesh were, respectively, 29%, 23%, 18%, and 9%. However, synthetic mesh was associated with a reoperation rate of 6.6%. Biological grafts had a reoperation rate of 3%, and surgery for mesh excision occurred in another 2.6%. For the posterior compartment, there were insufficient data to determine success rates. In comparison to native tissue repair, there was a trend toward lower failure rates with absorbable and nonabsorbable synthetic meshes but higher failure rates with biological grafts. However, there is much heterogeneity in biological grafts, and most studies evaluated by Jia et al. [5] used a porcine dermal graft.

Surgisis (Cook Surgical, Bloomington, IN) is a biological graft extracted from porcine small intestinal submucosa. In comparison to porcine dermal grafts, Surgisis has a higher collagen content, is acellular, and not cross-linked. In vivo, these characteristics result in graft resorption and replacement by host connective tissue. This may reduce long-term complications, but concerns have been raised about the durability of the resultant repair [6]. To date, very few studies have been published on the use of Surgisis for vaginal prolapse repair [7–10]. The aim of this study was to determine the success and complication rates of Surgisis in the treatment of anterior and posterior vaginal prolapse over a six-year period.

## 2. Surgical Procedures and Methods

From 2003 to 2009, 65 women with pelvic organ prolapse have been treated with Surgisis xenograft by four surgeons at the Flinders Urogynaecology Unit. Women treated with Surgisis were those considered at high risk of recurrence from traditional colporrhaphy or who had a recurrence after previous surgery. The treatments involved the anterior, apical, or posterior compartment, singularly or in combination, with the surgical technique adapted accordingly. Concomitant procedures, such as hysterectomy and urethral sling, were performed as clinically indicated.

For anterior repair, a midline vaginal incision is made from the bladder neck to the anterior fornix, followed by dissection from the pubocervical fascia at the bladder neck to the white line laterally and ischial spines superiorly. A protruding bulge, if present, is reduced with a purse string suture or midline plication. Next, a patient-tailored trapezoid-shaped graft is cut from a 10 × 7 cm sheet of four-layer Surgisis, partially rehydrated, and sutured to the boundaries of the anterior compartment to achieve a snug fit. The graft is first attached at the apex. With intact apical support, the graft is attached to the cervix or the vault scar. When apical support is deficient, it is sutured to the sacrospinous ligaments vaginally or to the uterosacral ligaments laparoscopically. The distal part of the graft is laterally attached to the white line at the level of the bladder neck and then transversally sutured to the bladder neck.

For the posterior compartment, repair starts with an inverted *T* incision beginning at the hymen and ending below the posterior fornix. The dissection is carried apically to the ischial spines, laterally to the pelvic side wall, and distally to the perineal body fascia. The apical arms of the graft are attached first. Then, the distal portion is trimmed and attached snugly to the perineal body fascia. Tacking sutures are placed to close the gap between the pelvic side wall and the lower half of the graft.

With combined anterior and posterior prolapse, a 20 × 7 cm four-layer Surgisis graft is cut and folded to create apical arms with anterior and posterior trapezoid extensions. The graft is attached superiorly with a nonabsorbable suture; elsewhere, a delayed absorbable suture is used. Redundant vaginal skin is trimmed and the wound closed with locking absorbable suture.

Cystoscopy is performed after anterior repair and a suprapubic catheter inserted under vision. Rectal examination is conducted after posterior repair to ensure absence of suture material in the rectum and exclude compression of the rectum.

Postoperatively, women are reviewed at six weeks, six months, annually up to three years, and then biannually. At each review, they are questioned about prolapse symptoms and bowel, bladder, and sexual function. POPQ assessments [11] are made at each visit. Objective success is defined as POPQ Stage 0 or 1 in all compartments and objective failure as Stage 2 or more in any compartment. Subjective success is defined as having no more than an asymptomatic bulge not protruding beyond the hymen and subjective failure as a recurrence of symptoms with no objective prolapse. Complications were classified and coded according to the International Urogynecological Association (IUGA) and International Continence Society (ICS) joint terminology and classification of the complications related to the use of prostheses and grafts [12].

The study was approved by the Flinders Ethical Committee as an audit activity.

## 3. Results

Table 1 summarizes the preoperative characteristics of our cohort. Of 65 women treated with Surgisis, 39 (60%) underwent an anterior and posterior repair. Sixteen (25%) had a posterior repair only and 10 (15%) an anterior repair only. Forty-four procedures (68%) involved attachment of Surgisis to either the sacrospinous or uterosacral ligaments. Other concomitant procedures included hysterectomy (37%), continence surgery (15%), hysteropexy (10%), and native tissue repair of another compartment (7.7%). Twenty-three of the total of 103 concomitant procedures were laparoscopically assisted, and none involved open abdominal surgery.

The average duration of the combined procedures was 102 minutes with a median of 150 minutes (range: 50–240). Median estimated blood loss was 300 mL (range: 20–1.550 mL). Median duration of hospitalisation was 4 days (range: 2–43), and mean followup was 75 weeks.

Major surgical complications occurred in 4 women (6.2%). Three had an estimated blood loss >1.000 mL or required transfusion (IUGA/ICS classification [12]: 7A.T1). One woman suffered a small bowel injury not recognised at the time of laparoscopy (5C.T1.S5). Other complications included 13 (20.0%) vaginal/pelvic infections treated with oral antibiotics (1C.T2.S1/S2), 11 (16.9%) urinary tract infections, 12 women (18.5%) required a suprapubic catheter for more than 7 days (4B.T2), and 7 (10.8%) reported either persistent or de novo dyspareunia (1B.T4.S2). There were no cases of graft exposure, erosion, rejection, or seroma formation.

The objective success rate (POPQ Stage 0 or 1 in all compartments) was 66% (43 of 65). Of 22 objective failures, 16 had an asymptomatic bulge above the hymen giving a subjective success rate of 92%. Three women (4.6%) had repeat surgery; two are planning further surgery; one remained symptomatic but declined further surgery.

Table 2 shows the success rate per compartment repaired, and Table 3 shows the sites affected by recurrence or subsequent prolapse. Of 10 women in the anterior only group, four developed further prolapse: one recurrence of cystocoele and three de novo rectocoeles. One cystocoele and one rectocoele are asymptomatic (Aa = −1, Ap = −1), and two women with rectocoele (Ap = 0) underwent fascial repair. Among 16 women in the posterior only group, four had a subsequent prolapse. Three, all remaining asymptomatic, developed a de novo cystocoele (Aa = −1, Ba = −1, Aa = +1). The affected site was not recorded for the other.

Fourteen of 39 women treated with both anterior and posterior Surgisis experienced further prolapse (Table 3). Nine involved the anterior compartment, two the vault and anterior wall, one the vault only, one the posterior compartment only, and, in one, the site was not documented. Three of the anterior compartment recurrences were symptomatic and underwent surgery with permanent polypropylene mesh.

Overall, the anterior recurrence rate was 12 of 49 (24.5%) and the same site reoperation rate (planned and performed) was 3 of 49 (6.1%). After anterior repair two women developed a rectocoele that required repair. The posterior recurrence rate was one in 55 (1.8%) with no reoperations required.

Table 4 displays the timing and site of subsequent prolapse regardless of the site repaired. There was a failure rate of 29% (16% objective and 13% subjective) in women followed up to a year. This rate did not change significantly for those followed up to five years, but absolute numbers are small. Symptomatic recurrences did not appear to increase over time.

## 4. Discussion

The quest for the ultimate prolapse repair continues unabated despite the availability of various prosthetic and graft materials. Although it is generally accepted that they result in lower short-term recurrence rates, especially in the anterior compartment, there are also substantial drawbacks. These include significant prosthesis- and graft-related complications, difficulty treating subsequent failures, and no demonstrated benefit on quality of life and sexual function [5, 13–15]. The ideal graft material would allow correction of vaginal anatomy whilst maintaining pelvic organ function. It would be biocompatible, inert, sterile, resistant to physical modification, of mechanical stress and shrinkage, readily available, inexpensive, with minimal risks of infection and rejection [16].

Biological grafts from other species (xenografts) have been used to repair hernias and pelvic organ prolapse for many years [17]. They are thought to reduce complications of erosion, fistula formation, and infection seen with permanent prosthetic material [6]. Several xenografts are currently used in vaginal reconstruction [17]. They differ in species of origin (bovine or porcine), site of harvest (pericardium, dermis, intestinal submucosa), sterilisation process, and cross-linking during manufacture.

Surgisis is an acellular, three-dimensional lattice of collagen, and extracellular matrix, not cross-linked, derived from the submucosa of porcine small intestine. Being acellular, Surgisis minimises risks of viral or prion transmission, inflammatory responses, rejection, and exposure [7, 16]. The absence of chemical cross-linking facilitates colonisation by host cells and avoids encapsulation and poor fixation at the graft-host interface, which could weaken the repair [6]. The matrix encourages host angiogenesis, connective tissue and epithelial differentiation and ingrowth, eventually replacing the graft with constructive connective tissue remodelling instead of scar tissue [18]. Graft resorption is believed to reduce long-term complications, such as graft exposure and dyspareunia. Concern remains, however, about the durability of the repair after remodelling [6].

Apart from congress abstracts, there are very few studies on the use of Surgisis in pelvic floor repair. These include one randomised controlled trial of women undergoing anterior compartment repair [8] and three retrospective comparative studies [7, 9, 10]. The randomised trial with 56 patients compared Surgisis with traditional anterior colporrhaphy [8]. The anatomical cure (POPQ Stage 0 or 1) at 12 months was 86.2% with Surgisis and 59.3% with conventional colporrhaphy [8]. Improvement in quality of life was similar in both groups. More intraoperative complications, mainly “excessive” blood loss without transfusion, occurred in the Surgisis group. There were no graft infections or exposures. In women treated for recurrent cystocoele, anterior colporrhaphy had a much higher failure rate than Surgisis (57.1% versus 14.3%). This supports the contention that women with recurrent prolapse are likely to have intrinsically weak support tissue or poor healing, benefiting most from augmented repair.

Chaliha et al. [7] reported on 28 women undergoing either colporrhaphy or Surgisis augmentation for anterior prolapse and found an improvement in objective measurements and quality of life at six months, but no difference at two years. However, lack of randomisation and small numbers limit interpretation of these data. A comparative study by Mouritsen et al. [9], with a median followup of three years, found better results with Surgisis than with anterior or posterior colporrhaphy, but the difference was not statistically significant. Reid and Luo [10] compared 108 bridging graft vaginal paravaginal repairs (89 using Surgisis) with 59 native tissue cystocoele repairs [10]. With bridging grafts, cystocoele persistence was reduced from 10.2% to 4.6% and late recurrences from 22.6% to 4.9% [10].

 A randomised trial reported by Paraiso et al. [19] is often considered relevant to the use of porcine implants in rectocoele repair [14]. It compared three different techniques, one of which included a porcine-derived graft (Fortagen). There was a significant improvement in quality of life and sexual function in all groups, but the Fortagen group had the highest anatomical failure rate. However, Fortagen is cross-linked and more prone to encapsulation and poor fixation at the graft-host interface than Surgisis.

 The current study presents 65 women followed for a variable time up to six years. Compared with other studies mentioned, our failure rate is slightly higher for the anterior compartment, but lower for the posterior compartment. Our results, relating predominantly to women with previous surgery or considered at increased risk of recurrence, support the use of Surgisis. Most subjective failures also occurred in the unrepaired compartment. The anterior only group had four “failures,” two of which were symptomatic rectocoeles that underwent repair. Of the four “failures” in the posterior only group, three were anterior and they remain asymptomatic. The development of de novo prolapse after repair could be due to a delayed manifestation of generally weak support tissue, undertreatment (prolapse in one compartment missed or masked by prolapse in another), or alteration in the vaginal axis, predisposing to later prolapse. More often than not, pelvic floor dysfunction is not confined to a single support structure [20]. Subclinical poor support in a particular compartment may thus become more manifest after correction of visible prolapse in another [20].

Thus far, Surgisis shows most promise in the treatment of recurrent rectocoele. At Flinders urogynaecology, fascial repair remains the primary approach for posterior prolapse. Surgisis is used for recurrences, and permanent mesh is used when both these procedures failed. For anterior prolapse, the approach is dictated by the integrity of the levator muscle [21], vaginal rugation, and vaginal sacculation. With clinically intact levators and no sacculation, anterior colporrhaphy is the primary approach. With avulsed levators, intact rugae, and no sacculation, laparoscopic paravaginal repair is preferred. The remaining cases of cystocoele are at high risk of recurrence with native tissue repair and require graft or prosthetic augmentation. In these circumstances, Surgisis is used for the primary repair and permanent mesh for recurrences.

In general, the frequency of recurrence after prolapse repair increases with time [22]. There were no indications to that effect in our study. This suggests that Surgisis, when effective, achieves a durable result, perhaps because the new connective tissue is stronger than the original or because the graft offers critical support while the new connective tissue gains in strength. However, 75% of the operations were performed between 2007 and 2009, resulting in only 10 with a followup of more than three years. This emphasises the well-recognized need for long-term followup after prolapse surgery, difficult to achieve as it may be, particularly when people remain asymptomatic.

## Figures and Tables

**Table 1:** Patient characteristics and preoperative assessments.

Characteristic	Number	Percent
Age in years (median, range)	66 (40–84)	
Weight in kg (median, range)	75 (48–110)	
Parity (median, range)	2 (0–4)	
Previous treatments		
Oestrogens	46	70.8
Physiotherapy	49	75.4
Pessary	38	58.5
Hysterectomy	28	43.1
Prolapse surgery	27	41.5
Prolapse stage (POPQ) [11]		
2	36	55.4
3	28	43.1
4	1	1.5
Presenting symptoms		
Vaginal lump	53	81.5
Bladder symptoms	37	56.9
Urgency	28	43.1
Stress	20	30.8
Hesitancy/retention	12	18.5
Recurrent infection	2	3.1
Bowel symptoms	26	40.0
Evacuation difficulty	22	33.8
Faecal/flatal incontinence	4	6.2
Pain	7	10.8
Dyspareunia	3	4.6
Back pain	2	3.1
Dragging discomfort	1	1.5

**Table 2:** Success and failure rates according to the compartment repaired.

Compartment repaired	No. of patients	Objective cure*	Subjective cure*	Failure
Anterior	10 (15.4%)	6	8	2
Posterior	16 (24.6%)	12	15	1
Both	39 (60.0%)	25	36	3
Total	65 (100%)	43	59	6

*Objective cure is defined as POPQ stage <2 at the last followup. Subjective cure refers to women with no symptoms, no bulge beyond the hymen, and happy with the result.

**Table 3:** Site of recurrence or subsequent prolapse according to the compartment repaired.

Site of subsequent prolapse	Compartment repaired		
	Anterior (*n* = 10)	Posterior (*n* = 16)	Anterior and posterior (*n* = 39)
Apical	—	—	3
Anterior	1	3	11
Posterior	3	—	1
Not specified	—	1	1
Total	4	4	14*

*Two women had a subsequent prolapse in two compartments (vault and anterior wall).

**Table 4:** Success and failure rates according to the duration of followup.

Duration of followup	No. of patients	Objective cure*	Subjective cure*	Failure
Up to 1 year	65	43	59	6
Up to 3 years	33	20	31	2
Up to 5 years	10	7	10	0
More than 5 years	3	2	3	0

*Objective cure is defined as POPQ stage <2 at the last followup. Subjective cure refers to women with no symptoms, no bulge beyond the hymen, and happy with the result.
